# Effect of auditory feedback differs according to side of hemiparesis: a comparative pilot study

**DOI:** 10.1186/1743-0003-6-45

**Published:** 2009-12-17

**Authors:** Johanna VG Robertson, Thomas Hoellinger, Påvel Lindberg, Djamel Bensmail, Sylvain Hanneton, Agnès Roby-Brami

**Affiliations:** 1 Laboratoire de Neurophysique et Physiologie, Université Paris Descartes, CNRS UMR 8119, 45 rue des St Pères, 75006 Paris, France; 2 Department of Physical Medicine and Rehabilitation, University of Versailles Saint-Quentin R. Poincaré Hospital, AP-HP, 104 Bd R. Poincaré, 92380 Garches, France; 3 Laboratoire de Neurobiologie des Réseaux Sensorimoteurs, Université Paris Descartes, CNRS UMR 7060, 45 rue des St Pères, 75006 Paris, France

## Abstract

**Background:**

Following stroke, patients frequently demonstrate loss of motor control and function and altered kinematic parameters of reaching movements. Feedback is an essential component of rehabilitation and auditory feedback of kinematic parameters may be a useful tool for rehabilitation of reaching movements at the impairment level. The aim of this study was to investigate the effect of 2 types of auditory feedback on the kinematics of reaching movements in hemiparetic stroke patients and to compare differences between patients with right (RHD) and left hemisphere damage (LHD).

**Methods:**

10 healthy controls, 8 stroke patients with LHD and 8 with RHD were included. Patient groups had similar levels of upper limb function. Two types of auditory feedback (spatial and simple) were developed and provided online during reaching movements to 9 targets in the workspace. Kinematics of the upper limb were recorded with an electromagnetic system. Kinematics were compared between groups (Mann Whitney test) and the effect of auditory feedback on kinematics was tested within each patient group (Friedman test).

**Results:**

In the patient groups, peak hand velocity was lower, the number of velocity peaks was higher and movements were more curved than in the healthy group. Despite having a similar clinical level, kinematics differed between LHD and RHD groups. Peak velocity was similar but LHD patients had fewer velocity peaks and less curved movements than RHD patients. The addition of auditory feedback improved the curvature index in patients with RHD and deteriorated peak velocity, the number of velocity peaks and curvature index in LHD patients. No difference between types of feedback was found in either patient group.

**Conclusion:**

In stroke patients, side of lesion should be considered when examining arm reaching kinematics. Further studies are necessary to evaluate differences in responses to auditory feedback between patients with lesions in opposite cerebral hemispheres.

## Background

Less than half of stroke patients regain functional use of their arm [1] making recovery of upper limb function a major aim of stroke rehabilitation. Studies using movement analysis techniques have shown alterations in movement patterns following stroke, including: decreased velocity, alterations in the shape of the velocity curve, loss of smoothness and loss of inter-joint coordination [2,3]. These impairments may result as a direct consequence of the lesion however secondary compensatory strategies are also observed [2].

Rehabilitation aims to improve function but training at the impairment level may be necessary so that patients reach their full potential [4]. Analysing movement kinematics may allow identification of important movement parameters for training. The addition of augmented feedback may help to improve movement performance and thus complement conventional therapy.

Augmented feedback is the addition of a feedback not normally present in the environment, as distinct from intrinsic feedback which refers to a person's own sensory-perceptual information that is available as a result of the movement being performed. Feedback may be given to enhance knowledge of how the task is performed (knowledge of performance, KP) or regarding goal achievement (knowledge of results, KR) [5]. Since following a stroke, intrinsic feedback mechanisms are frequently compromised, the provision of extrinsic feedback may be beneficial [6] and different types of feedback (KP or KR) may be used depending on the aims of rehabilitation. In a review, Van Vliet and Wulf [6] concluded that although evidence for the use of augmented feedback in stroke rehabilitation is lacking, auditory and visual feedback appear to be beneficial. Certain criteria appear to affect the effectiveness of the feedback such as: (i) the number of trials with feedback (less than 100% of trials is more effective) and (ii) if the feedback induces an external focus (i.e., that the patient increases attention to target position etc) [6]. It has been suggested that the provision of specific impairment-related feedback may be able to elicit motor learning and to affect motor recovery even in chronic stroke [4]. KP has been shown to be more effective than KR for generalisation of learning to different tasks in chronic stroke patients [7,8].

Few studies have evaluated the use of auditory feedback to guide upper limb movements in stroke patients. Maulucci et al. [9] used auditory feedback which informed subjects of the deviation of their hand from the ideal path by use of a tone which was emitted if the hand strayed out-with the 'normal reach zone'. The frequency of the tone increased with distance from the normal zone. After 6 weeks of training, hand path was significantly closer to the normal path and changes in movement direction were significantly decreased in the experimental group. The control group, who practiced the same movements with no feedback, showed fewer improvements.

Huang et al. [10] evaluated a novel musical feedback relating to movement smoothness in two stroke patients. The feedback consisted of a musical phrase (piano) which was only recognisable if hand motion was smooth. Compensation by use of trunk movements was discouraged by interference of other instruments (violins) which occurred if the trunk was flexed beyond a predetermined distance. In this small pilot study, they found that, when the musical feedback was added to the visual feedback provided by means of virtual reality, hand trajectories became smoother.

In order to further study the potential of auditory feedback during upper limb movements after stroke we developed a method that delivered auditory feedback during reaching movements. In this pilot study we wanted to investigate whether the auditory feedback could modify the quality of the hand trajectory during a reaching movement in stroke patients. We chose to provide the auditory feedback during the movement for several reasons: (i) it can be delivered easily online; (ii) it can induce an external focus to movement, (iii) since adding auditory feedback might be complementary without interfering with normal visual or proprioceptive feedback processes. Two types of auditory feedback during arm reaching were developed: (i) simple feedback, which gives information regarding the distance (by increasing or decreasing volume); and (ii) spatial feedback, which gives information regarding the direction of the target (by spatial distribution of sound in either ear). The feedback was provided on all trials since the aim of this study was not to evaluate learning but to test the immediate effect of the feedback. Work within our team on sensory substitution (visual to auditory) demonstrated that healthy subjects could use an auditory feedback to explore their environment with no prior knowledge of the characteristics of the feedback [11]. This suggests that the human brain is able to directly extract spatial information from natural sound sources. We therefore hypothesized that this kind of feedback could be used directly by patients to obtain information about the hand trajectory and that such feedback may improve the trajectory in stroke patients.

In this pilot study we were also interested in investigating whether the effects of auditory feedback differed depending on side of stroke lesion. In stroke patients, reaching for targets of different distances with the ipsilesional arm results in different modulations of acceleration amplitude and duration, according to side of brain lesion [12]. This suggests that kinematics during reaching with the paretic arm (contralesional) may also differ depending on side of the stroke. However, to our knowledge, this has not been tested directly. We also considered it likely that side of the lesion would influence response to auditory feedback since auditory processing may be lateralised in the brain [13]. Therefore we also postulated that performance during reaching for a target and effect of auditory feedback would differ depending on the side of hemisphere damage.

## Method

### Subjects

Ethical approval for the study was obtained and patients (or their family in one case) gave informed consent. Patients were included if they were over 18 years and had hemiparesis of vascular origin with sufficient recovery to complete the task. They were excluded if they had multiple cerebral lesions, acute algoneurodystrophy, cerebellar involvement, comprehension deficits preventing participation in the experiment or hearing deficits. Hearing deficits were assessed with a home-made hearing test (validated informally in 10 healthy subjects). This involved listening to 12 tones ranging from 125 Hz to 15000 Hz played in each ear via headphones. The volume was set to a comfortable level for each subject. Subjects were asked in which ear they heard the tone. Subjects were excluded if they had less than 10/12 correct responses in each ear. A total of 16 hemiparetic patients were included in the study, eight with left hemisphere damage (LHD) and eight with right hemisphere damage (RHD) following a first ischemic or hemorrhagic stroke with cortical and/or subcortical lesions (Table 1). In the LHD group, three subjects were female and the average age was 57 years (range 46-79). In the RHD group, two were female and average age was 48 years (range 28-78). There was no statistically significant difference between the ages of the two groups. Subjects used their hemiparetic arms for the experiment. All the LHD patients were right handed and so used their dominant hands for the experiment. 6 out of 8 RHD patients were right handed and therefore used their non-dominant hands for the experiment while the 2 left-handed RHD patients used their dominant hands.

**Table 1:** Subject details

Subject	Age	Sex	Time since onset (mths)	Lesion site	Type of stroke	Dominant hand	Neglect/Aphasia/Apraxia/
**1**	28	M	11	R deep MCA	Isch	Left	nil
**2**	78	M	1	R paraventricular	Isch	Right	nil
**3**	32	M	27	R sup + deep MCA	Isch	Right	nil
**4**	33	F	4	R capsulo thalamic	Haem	Right	nil
**5**	55	F	3	R Pontine	Isch	Left	nil
**6**	53	M	1	R sup MCA	Isch	Right	nil
**7**	66	M	6	R parieto-occipital	Haem	Right	Mild neglect
**8**	35	M	3	R fronto-parietal focal	Haem	Right	Mild neglect
**9**	69	M	5	L MCA	Isch	Right	Apraxia (++) Mild neglect Aphasia (1)
**10**	52	M	1.5	L sup + deep MCA	Isch	Right	Apraxia(++) Aphasia (2)
**11**	58	M	6	L anterior cereb + MCA	Isch	Right	Apraxia(++) Mild neglect Aphasia (1)
**12**	79	F	3	L capsulo-thalamic	Haem	Right	nil
**13**	53	F	5	L MCA	Haem	Right	Apraxia(++) Aphasia (3)
**14**	50	F	9	L choroidial artery	Isch	Right	nil
**15**	46	M	2	L sup+ deep MCA	Isch	Right	Aphasia (3)
**16**	52	M	1.5	L post lenticulaire	Isch	Right	Aphasia (5)

Abreviations: M = male, F = female, R = right, L = left, Mca = middle cerebral artery, sup = superficial, Isch = ischemic, Haem = haemorrhagic, Apraxia(+) = mild, Apraxia(++) = interferes with ADL, Aphasia score according to the Boston Diagnostic Aphasia Examination.

10 healthy subjects also performed the task in order to have reference values of hand kinematics with which to compare the patients. No auditory feedback was provided to the healthy subjects as it was assumed that it would have no effect on 'normal' movements. All healthy subjects were right handed and had no neurological or orthopaedic pathology of the upper limb. Mean age was 41 years (range 25-69). Healthy subjects performed reaching movements with their right hands.

### Clinical evaluation

Scores of routine clinical tests were used to compare the level of impairment and functional ability across patient groups: Action Research Arm Test (ARAT) [14], Box and Block test [15] and Barthel Index [16] (Table 2). These tests were all carried out by the patient's individual therapist, independently of the study. The ARAT measures arm and hand function, the Box and Block tests dexterity and gross motor coordination and the Barthel Index is measure of functioning in basic activities of daily living. In Tables 1 and 2, patients are ranked according to level of impairment as measured by the ARAT. There were no significant differences in clinical scores between LHD and RHD patient groups for ARAT (p = 0.83), Box and Block (p = 0.25) or Barthel Index (p > 0.99).

**Table 2:** Clinical scores

RHD	ARAT	Box and Block	Barthel	LHD	ARAT	Box and Block	Barthel
**1**	9	3	100	**9**	15	0	90
**2**	27	18	65	**10**	18	9	95
**3**	28	8	85	**11**	33	17	25
**4**	38	16	95	**12**	36	74	85
**5**	43	33	70	**13**	49	30	90
**6**	51	28	95	**14**	52	41	90
**7**	52	29	90	**15**	56	53	100
**8**	57	34	100	**16**	57	51	100
**MEAN (SD)**	38.1 (16.1)	21.1 (11.7)	87.5 (13.4)	**MEAN (SD)**	39.5 (16.7)	34.4 (25.0)	84.4 (24.6)

Symptoms such as aphasia, apraxia and neglect were also noted (Table 1). Aphasia was scored according to the Boston Diagnostic Aphasia Examination [17]. This is a scale from 0 to 5 on which a score of 0 indicates no intelligible expression or oral comprehension and 5 indicates a hardly noticeable disability which cannot be objectively measured. Apraxia was scored as mild or severe according to if it interfered or not with activities of daily living (ADL). Neglect was scored using the Bell Test [18]. None of the patients found to have neglect actually neglected more than 10 bells; this was considered as mild neglect.

### Protocol

Patients were asked to carry out reciprocal pointing movements with their hemiparetic arm in three conditions: no feedback, simple auditory feedback and spatial auditory feedback. The two types of feedback were tested on different days in order to 'washout' any effect one might have on the other. On each day, a no feedback condition was also carried out as a control. The order of the sessions was randomized and the randomization procedure was stratified according to left or right hemiparesis. Patients could be randomized into one of four 'session orders' (Table 3). In each 'session order', there were two LHD and two RHD patients.

**Table 3:** Four possible session orders

Day 1		Day 2	
Session1	Session2	Session3	Session4
No feedback	Simple feedback	No feedback	Spatial feedback
No feedback	Spatial feedback	No feedback	Simple feedback
Simple feedback	No feedback	Spatial feedback	No feedback
Spatial feedback	No feedback	Simple feedback	No feedback

### Experimental set up and task

The task consisted of making three reciprocal movements to each of nine targets (a total of 27 movements per condition). The starting position was with the hand on the abdomen, the elbow flexed at approximately 90° and the shoulder in approximately 0° flexion and slight abduction. The instruction given was to place the palm of the hand on the target and return the hand to the abdomen three times consecutively at a comfortable speed. So as not to interfere with natural movements, the starting position of the arm was not checked during the three consecutive movements. Patients were, however, instructed before beginning to place their hand on the same part of the abdomen after each movement. Targets were presented in a standardized order.

**Figure 1:**
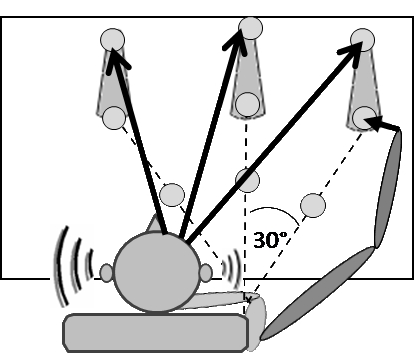
**Position of subject and targets and pictorial indication of spatial sound for one target**. The position of the targets relative to a subject with right hemiparesis is shown. Six targets were positioned on the surface of the table and three were on a removable support. Targets were arranged on three lines (saggital and 30° to the left and to the right). Thick arrows indicate movement distance. The intensity and frequency of the sound in each ear depended on the direction and position of the hand relative to the target.

The nine targets were positioned on a table in front of the subject. Target distance was adjusted for each patient, depending on arm length, measured from the acromion to the centre of the palm (since the palm was the 'working point'). This measurement was used to position the targets for each individual. Six targets were positioned on the surface of the table: 3 close (60% arm length), and 3 far (90% of arm length), and three were high (17 cm above the corresponding far target, on a removable support). Targets were arranged on three lines, one in the saggital plane, in line with the subject's shoulder, and the other two on lines angled 30° to the left or right of the saggital line (Figure 1). The movement distance to reach the internal targets was shorter than for external targets because of the starting position of the hand (as indicated by the thick arrows in Figure 1). Target positions for left-sided hemiparetic patients were the mirror image of those of the right-sided patients.

Patients were comfortably seated on a chair adjusted so that the table was approximately level with the navel. Target position and chair height and position were marked so as to ensure that the same positions were used during the two visits. Patients wore headphones for the feedback delivery.

### Data collection

Recordings were made using an electromagnetic Polhemus system (acquisition frequency = 30 Hz) with 4 sensors and an emitting source fixed underneath the table. The sensors were placed on the sternum (just below the manubrium), acromion, upper arm (at the level of the deltoid insertion) and on the dorsum of the hand, on the middle of the third metacarpal bone. The Polhemus system gives displacement data and Euler angles (azimut, elevation, roll) for each sensor. Only data from the hand sensor will be presented here. A small splint was used to prevent wrist motion.

### Auditory feedback

An OpenAl software library was used to create an audio-motor coupling. The sound was a complex "buzzing" sound similar to a fly whose envelop varied between 100-3000 Hz). The data of the Polhemus sensor fixed to the hand were processed on line to modulate the sound heard in the headphones. Two types of feedback were tested; simple and spatial. In the case of the simple feedback, the volume increased as the hand approached the target. For the spatial feedback, as well as increasing volume with proximity to the target, the sound perceived depended on 3D orientation of the hand relative to the target. The spatial auditory model simulates binaural spatial cues like interaural level differences and interaural time differences [19]. In the horizontal plane, the sound was equally balanced if the hand pointed directly towards the target. If the hand was not orientated directly towards the target, the sound was 'muffled' in one ear in the same manner as it would be in the right ear when listing to a radio on the left side of the body (Figure 1).

Patients were not informed of the particularities of the feedback. They were simply told that they would hear a sound. Before each feedback session, they were given a chance to explore the workspace with the feedback switched on for as long as they desired (usually less than one minute).

### Data analysis

Velocity curves of the hand sensor were calculated by derivation of the displacement data. A Labview routine was used to automatically detect the beginning and end of movements (with a threshold of 0.05 m/s), these were then visually checked. Only movements towards the targets were analysed and not the return movements.

Three kinematic variables were analyzed, all relating to hand trajectory: peak movement velocity, movement smoothness (number of velocity peaks) and global trajectory curvature (curve index, calculated as the ratio between the actual hand path length and the direct length from the starting to finishing points [20]).

### Statistics

Because the data were not normally distributed, non parametric tests were used. For multiple, paired data, a Friedman test was used. If the result was significant, the Wilcoxon sign test was used to determine which pairs differed. For comparison of unpaired data, the Mann-Whitney test was used. p < 0.05 was taken as significant in each case.

## Results

### Movement kinematics

**Figure 2:**
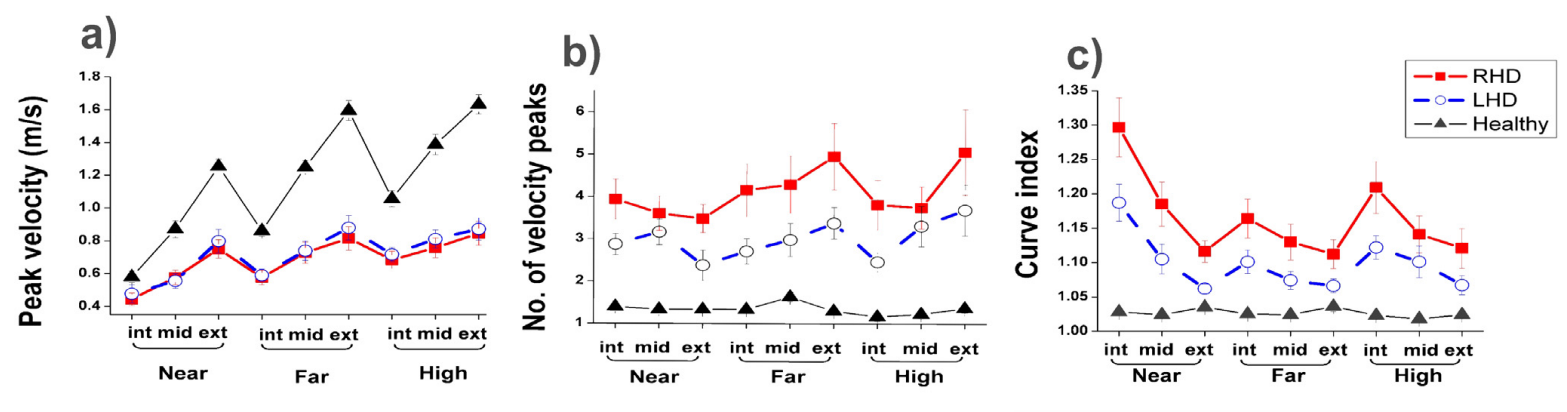
**Comparison of kinematic variables between subject groups**. Mean values and standard errors are presented (healthy group = black triangles, RHD = red squares, LHD = blue open circles). All data are from the 'no-feedback' condition. a) peak velocity b) number of velocity peaks c) curve index. Int = internal; mid = middle; ext = external. Kinematic performance of healthy subjects was significantly better than patients and performance of LHD patients was significantly better than RHD patients.

Graphs for the 3 kinematic variables analysed are provided in Figure 2a, b, c. Data for healthy subjects and the two patient groups (RHD and LHD) are superimposed. All data displayed in these figures was collected in the no-feedback condition. Comparison of healthy subjects and patients showed that peak hand velocity was much greater in healthy subjects (p < 0.0001) and the number of velocity peaks (p < 0.0001) and curve index (p < 0.0001) were much lower. Healthy subjects also displayed much less variability. Peak hand velocity was scaled according to target distance in healthy subjects as well as in patients with RHD and LHD, although to a lesser extent in the patient groups.

Targets were grouped into 'near', 'far' and 'high' (distance condition) and 'internal', 'middle' and 'external' (direction condition). Peak velocity increased significantly between near and far (p < 0.0001) and near and high targets in healthy subjects and both patient groups (p < 0.0001) and also between high and far targets in the LHD group (p = 0.005). Peak velocity was significantly higher for external targets compared with internal (p < 0.0001), and middle (p < 0.0001) in healthy subjects and both patient groups (Figure 2a). There were no significant differences between the target conditions for the number of velocity peaks in healthy subjects or either of the patient groups (Figure 2b). Trajectories were significantly less curved for high compared with near targets (p = 0.003) and high compared with far targets (p = 0.02) in healthy subjects and for far compared with near targets for both RHD (p = 0.006) and LHD groups (p = 0.0003) (Figure 2c). In the LHD group, trajectories to far targets were also significantly less curved than to high targets (p = 0.002). Trajectories were significantly more curved for external compared to middle targets in healthy subjects (p = 0.02) but were less curved in both patient groups for external compared to internal targets (RHD p < 0.0001, LHD p < 0.0001)

LHD and RHD groups were compared by grouping all targets together. Peak velocity did not differ between groups (p = 0.85). The RHD group had significantly more velocity peaks than the LHD group (p = 0.004) and significantly greater trajectory curvature (p < 0.0001).

### Effect of feedback

In order to compare the effect of the simple and spatial feedbacks on movement parameters, the percentage difference between the auditory feedback sessions and the control (no feedback) sessions carried out on the same day was calculated for each variable. The percentage difference relating to the simple feedback was then compared with that relating to the spatial feedback. No significant differences were found for any of the parameters analysed. Mean and SD data for each kinematic variable in the different feedback conditions are presented in table 4. Each subject was asked to describe the nature of the sound after the sessions. Only one subject was aware of the spatial nature of the feedback, he was a musician (Subject 3).

**Table 4:** Mean (SD) values of parameters evaluated in each condition. NF = no feedback

		Same day						Same day					
		NF			Simple			NF			Spatial		
**LHD**	**Peak velocity**	0.73	±	0.28	0.68	±	0.25	0.70	±	0.23	0.64	±	0.23
	**N° vel. peaks**	2.87	±	1.42	3.54	±	2.10	3.11	±	1.59	3.53	±	2.04
	**Curve index**	1.10	±	0.08	1.12	±	0.09	1.10	±	0.08	1.11	±	0.10
**RHD**	**Peak velocity**	0.70	±	0.27	0.69	±	0.26	0.67	±	0.24	0.72	±	0.27
	**N° vel. peaks**	4.36	±	2.66	3.92	±	2.23	3.85	±	2.23	3.31	±	1.73
	**Curve index**	1.17	±	0.13	1.15	±	0.13	1.16	±	0.13	1.15	±	0.12

**Figure 3:**
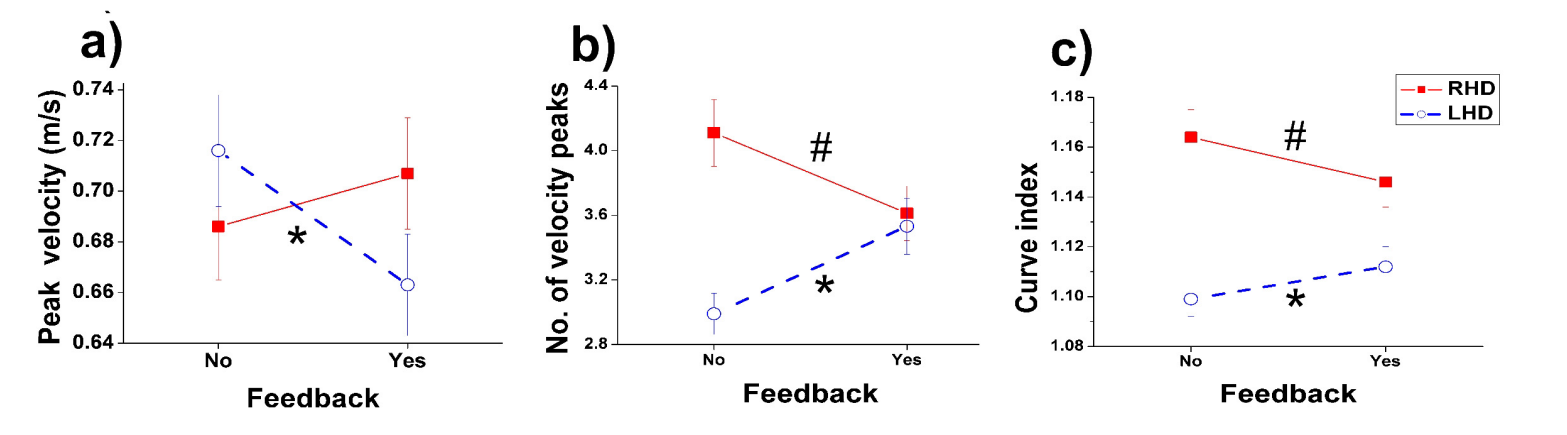
**Comparison of kinematic variables with and without auditory feedback**. Mean values and standard errors are presented (RHD = red squares, LHD = blue open circles). a) peak velocity b) number of velocity peaks c) curve index. * indicates a significant difference between conditions for LHD group, # indicates a significant difference between conditions for RHD group. The presence of feedback improved performance in the RHD group and degraded the LHD group.

**Figure 4:**
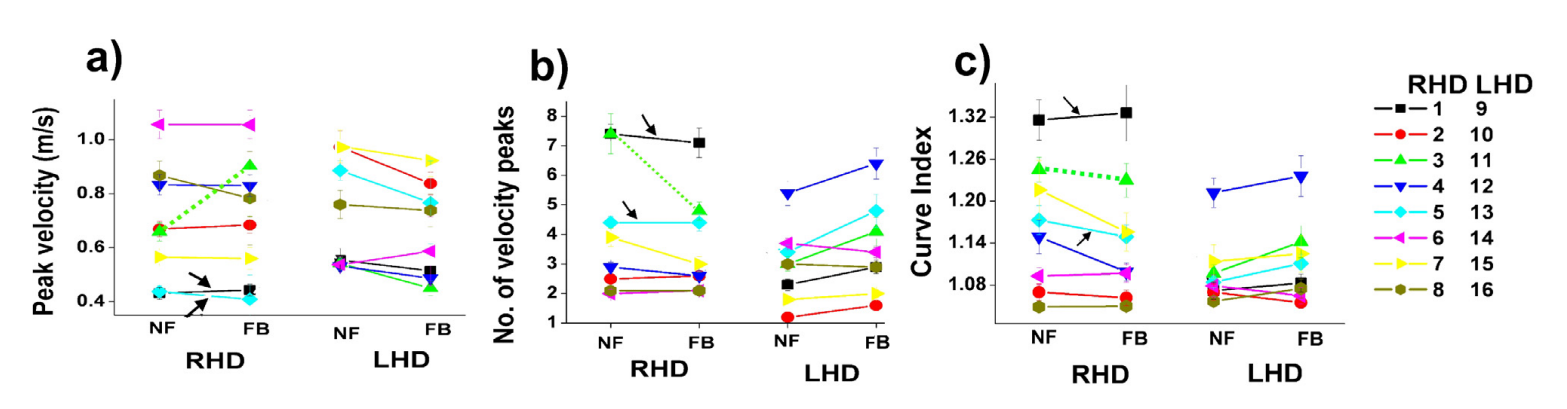
**Individual kinematic data in RHD and LHD patient groups with and without feedback**. Comparison of condition without (NF = no feedback) and with feedback (FB = feedback). Each shape represents an RHD and an LHD subject (see ID on Figure legend). Arrows indicate the two left handed patients (subjects 1 and 5) and the dashed green line indicates one patient who responded differently from his group (subject 3).

Because there was no difference between the effects of the different types of feedback, the data were pooled into a 'feedback' condition and a 'no-feedback' condition for further analysis (Figure 3a, b, c). The addition of auditory feedback had different effects on LHD and RHD groups. Although peak velocity did not change significantly, a generally beneficial effect was noted in the RHD group with a significant decrease in the number of velocity peaks (p = 0.0003) (Figure 3b) and a significant decrease in trajectory curve index (p = 0.005) (Figure 4c). The opposite effect was noted for the LHD group: significant decrease in peak velocity (p < 0.0001) (Figure 3a), significant increase in the number of peaks in the velocity curve (p < 0.0001) (Figure 3b) and significant increase in curve index (p = 0.02) (Figure 3c).

We also examined individual responses to feedback to check if patients in each group followed the same tendencies (Figure 4). It appeared that the kinematic performance of the majority of LHD patients did worsen in the presence of feedback while in the RHD group, patients changed less except for one subject who was a musician (subject 3 denoted by green dotted lines in Figure 4) who had a much greater response to the feedback than the others. When statistical analysis was repeated without this patient, the effect of feedback on peak velocity remained non-significant (p = 0.13), the decrease in the number of velocity peaks was no longer significant but tended towards significance (p = 0.067) and the decrease in curve index remained significant (p = 0.005) (results in figures and tables include all patients).

### Dominant versus non-dominant hand

Since two of the patients in the RHD group were left-handed, we examined individual data in order to determine if the differences in response to feedback between the two groups were the result of individual differences related to the use of dominant versus non dominant hand or left versus right hemiparesis. The two left handed patients in the RHD group (marked with arrows in Figure 4) used their dominant hands and had movement parameters at the lower level of the group, but the effect of feedback appeared similar to that observed in the other RHD patients (no or little change) while movement parameters of most LHD patients worsened (Figure 4).

## Discussion

The main findings of this pilot study were that, despite having similar functional capacity and similar movement velocity, the RHD and LHD patients exhibited differences in movement smoothness and curvature and showed opposite responses to the feedback. Patients in the RHD group showed a consistent improvement in curvature with the addition of auditory feedback whereas patients in the LHD group showed a consistent deterioration of all movement parameters.

### Kinematic characteristics

We observed low peak velocities, lack of smoothness and increased curvature of the hand trajectories of the stroke patients compared with the healthy subjects. This is in agreement with previous studies [2,3]. Peak hand velocity was scaled with target distance in both healthy subjects and patients consistent with previous reports [21]. Peak velocity was significantly higher for external compared with internal targets in healthy subjects and patients. This is likely to be related to the fact that the movement distance was greater to the external targets but it has also been shown in healthy subjects that hands move faster in their own hemispace [22,23]. In the patient groups, the curve index was significantly lower for external than internal targets while the opposite was true for the healthy subjects. Desmurget et al. [24] reported similar results in healthy subjects, the curve index increased as targets became more external. Perhaps this indicates a particular control problem for hemiparetic patients in the internal part of the workspace. Indeed, Levin [3] found greater disruption in shoulder-elbow coordination in hemiparetic patients for internal targets compared with external targets.

To our knowledge, this is the first study to compare kinematics of the contralesional hand between left and right hemispheric lesions. We found that smoothness and curvature of the hand trajectory were different between the LHD group and the RHD group. The LHD group had generally less curved and smoother movements than the RHD group, denoting better kinematic movement quality. This difference cannot be explained by different levels of impairment between the groups since clinical scores and peak hand velocity were not significantly different between the groups. It is possible that the kinematic differences between our groups reflect some specialization of the lesioned cerebral hemisphere. We speculate that movement smoothness and curvature may be predominantly controlled in the right hemisphere. This is indirectly supported from findings in studies comparing hand performance in healthy subjects [22,25] and ipsilesional hand performance between left and right hemispheric lesions [12,26,27]. The left hemisphere has been linked with an open-loop form of control [26], specialized in the control of limb dynamics [28,29] and temporal processing [30]. The right hemisphere is believed to function in a closed loop, specialised in the control of on-line visual processing [26] and final limb posture. Right hemisphere damage has been found to reduce final position accuracy of the right hand while it does not reduce accuracy of the left hand [12]. It seems likely that the kinematic differences we found between patients with LHD and RHD reflect differences in hemispheric specialization. Further study is warranted to confirm this.

The presence of apraxic patients within the LHD group may be a confounding factor in this study. However, kinematic errors tend to increase in apraxic patients with task difficulty (decreased visual feedback and target size) [31] while our task was simple requiring little precision. Hermsdörfer et al. [32] also showed that errors linked to apraxia were not correlated with kinematic errors. Also, our LHD patients, including apraxic patients, demonstrated better kinematics than the RHD group. Therefore we consider it unlikely that presence of apraxia could completely explain the kinematic differences found between patient groups in this study.

### Effect of feedback

The addition of auditory feedback had the opposite effect in each group. The group mean for each variable analyzed improved in the RHD group while it deteriorated in the LHD group when the feedback was added.

Visual analysis of individual responses to the feedback (Figure 4) showed that one subject in the RHD group had a much greater response than other patients in his group. This patient was a musician and therefore had highly developed auditory function. However, even when he was excluded from the analysis, the results remained essentially the same (although the decrease in number of velocity peaks then only tended to significance). Although these improvements were not consistent across all parameters, they suggest that auditory feedback could be a useful tool to improve movement kinematics in RHD patients. However, this remains to be tested with different types of feedback.

It is known that patients with aphasia can also have deficits in non-verbal sound processing [33] and five of our eight LHD patients had receptive aphasia which may explain why the auditory feedback was disruptive in this group. The particular characteristic of our feedback was the sensation of motion. Several studies have demonstrated specific deficits in motion detection or auditory spatial localisation following right hemisphere lesions which are linked with neglect [34]. However, on comparison of deficits arising from lesions in opposite hemispheres Adriani et al. [35] found no difference in sound localisation or motion perception deficits between patients with LHD or RHD. It is not possible to ascertain if the detrimental effect of the feedback was linked to the degree of aphasia because of we did not quantify the degree of aphasia and too few LHD patients were included for further subgroup analysis. Degree of aphasia may thus be a confounding factor in this study and further investigation into the relation between degree of aphasia and reaching kinematics is indicated.

Degree of spatial attention deficits may be another possible explanation for different effects of feedback depending on side of lesion. Our auditory feedback task could be considered similar to a dual task since patients were required to perform reaching movements while listening to feedback. This may have had greater attentional requirements than carrying out reaching movements alone. Hyndman et al. [36], however found that patients with RHD tended to have worse divided attention than LHD so the dual task hypothesis does not seem to explain the difference in our groups. However in the same study, the LHD exhibited slightly worse auditory selective attention (patients were asked to count low tones while ignoring high tones) at discharge than RHD (the differences were trends). Perhaps the difference between our groups is therefore related to the processing of the feedback itself. Some evidence suggests that the left-hemisphere may be superior with regard to on-line feedback processing during goal-directed movements although this evidence tends to come from studies using visual feedback [37]. Lesions of the left hemisphere may therefore disrupt feedback processing capacity.

In short, our results demonstrated a difference in the effect of the auditory feedback depending on side of lesion. It is not possible, however, to determine if this is related to a difference in processing of auditory information or the fact that each hemisphere has a different role in movement control or an interaction of the two factors.

### Lack of effect of spatial feedback

Our group previously showed that healthy subjects are able to use spatial feedback to locate unseen static virtual objects using spatial feedback [38]. We therefore hoped that this unusual feedback could be integrated online in a similar manner to visual feedback [39] and guide hand orientation in patients. We suggest three explanations for the lack of effect of the spatial nature of the feedback.

1) It is possible that the directional component of our spatial feedback was either too complex or too subtle to be integrated implicitly in patients with cerebral lesions. However, a similar type of spatial feedback was successfully used for sensory substitution in patients with vestibular disorders [40] but in this study the patients were aware of the nature of the feedback and they did not have cerebral lesions.

2) Perhaps auditory feedback is poorly adapted for spatial guidance of the hand. It has previously been shown that subjects rely more on visual than proprioceptive feedback and adjust movement trajectories so as to ensure visually constant movements [41]. We allowed use of vision since we wanted to assess an *augmented *feedback, not a substitution. However, the auditory feedback may have been superfluous if patients gained sufficient intrinsic feedback (visual and proprioceptive). Auditory feedback may be better adapted for temporal guidance, such as in that developed by Huang et al. [10] (described in the introduction), since temporal parameters are well coded in the auditory cortex while the visual cortex codes predominantly spatial information.

3) Although moving sounds can be detected by a single hemisphere, for accurate discrimination of sound motion, interaction between both hemispheres may be necessary for the interpretation of interaural differences [34]. In patients with cerebral lesions of one hemisphere, capacity to process moving sounds might be reduced. Indeed, only one of the sixteen patients actually became aware of the spatial nature of the sound, he was a musician.

## Conclusion, limitations and perspectives

Studies of stroke patients usually restrict subject inclusion to right handed patients with left hemisphere damage or they do not make comparisons between patient groups. Until now, no study has compared the effect of feedback in patients with left versus right hemisphere damage. We found that patients with left hemisphere damage made smoother, less curved movements than patients with right hemisphere damage despite having a similar level of impairment and peak hand velocity. The kinematic performance of the LHD group was degraded by the presence of auditory feedback while that of the RHD group was not. These results demonstrate a need for thorough investigation of differences in motor abilities in a variety of environments and conditions between patients with left and right hemisphere lesions before developing complex rehabilitation methods such as virtual reality.

It is important to note, however, that the small sample size and heterogenous population, including patients with neuropscychological deficits mean that our results must be interpreted with caution. Equally, the presence of 2 left-handed patients within the RHD group may have confounded the results although the role of each hemisphere may be independent of hand preference [22,25].

In stroke patients, auditory feedback may not be suitable for the provision of knowledge of performance when discrimination of features of the sound is necessary. The manner in which different aspects of sound are processed is not yet fully understood and the presence of cerebral lesions may render perception of changes in sound difficult for patients. Perhaps visual feedback is a more appropriate mode of provision of knowledge of performance of spatial aspects during hand movement while auditory feedback may be better adapted for the provision of temporal information or knowledge of results or to warn of errors. Further study is indicated in the use of auditory feedback in stroke patients.

## Competing interests

The authors declare that they have no competing interests.

## Authors' contributions

JVGR and ARB participated in the conception and design of the protocol, analysis and interpretation of data and drafting the article, TH and SH participated in the conception and design of the protocol and created the feedback, PL and DB were involved in data interpretation and helped to draft the article. All authors gave final approval of the version submitted.

## References

[B1] Jorgensen HS Nakayama H Raaschou HO Vive-Larsen J Stoier M Olsen TS Outcome and time course of recovery in stroke. Part I: Outcome. The Copenhagen Stroke Study Arch Phys Med Rehabil 1995 76 399 405 10.1016/S0003-9993(95)80567-2 7741608

[B2] Roby-Brami A Feydy A Combeaud M Biryukova EV Bussel B Levin MF Motor compensation and recovery for reaching in stroke patients Acta Neurol Scand 2003 107 369 381 10.1034/j.1600-0404.2003.00021.x 12713530

[B3] Levin MF Interjoint coordination during pointing movements is disrupted in spastic hemiparesis Brain 1996 119 281 293 10.1093/brain/119.1.281 8624689

[B4] Michaelsen SM Dannenbaum R Levin MF Task-specific training with trunk restraint on arm recovery in stroke: randomized control trial Stroke 2006 37 186 192 10.1161/01.STR.0000196940.20446.c9 16339469

[B5] Schmidt R Wrisberg C Motor learning and performance 2004 Leeds, England: Human Kinetics

[B6] van Vliet PM Wulf G Extrinsic feedback for motor learning after stroke: what is the evidence? Disabil Rehabil 2006 28 831 840 10.1080/09638280500534937 16777770

[B7] Cirstea CM Ptito A Levin MF Feedback and cognition in arm motor skill reacquisition after stroke Stroke 2006 37 1237 1242 10.1161/01.STR.0000217417.89347.63 16601218

[B8] Cirstea MC Levin MF Improvement of Arm Movement Patterns and Endpoint Control Depends on Type of Feedback During Practice in Stroke Survivors Neurorehabil Neural Repair 2007 21 398 411 10.1177/1545968306298414 17369514

[B9] Maulucci RA Eckhouse RH Retraining reaching in chronic stroke with real-time auditory feedback NeuroRehabilitation 2001 16 171 182 11790902

[B10] Huang H Interactive multimodal biofeedback for task-orientated neural rehabilitation27th Annual International Conference of the IEEE Engineering in Medicine and Biology Society Shanghai 2005 2547 2550 10.1109/IEMBS.2005.1616988 17282757

[B11] Auvray M Hanneton S O'Regan JK Learning to perceive with a visuo-auditory substitution system: localisation and object recognition with 'the vOICe' Perception 2007 36 416 430 10.1068/p5631 17455756

[B12] Schaefer SY Haaland KY Sainburg RL Ipsilesional motor deficits following stroke reflect hemispheric specializations for movement control Brain 2007 130 2146 2158 10.1093/brain/awm145 17626039 PMC3769213

[B13] Alain C He Y Grady C The contribution of the inferior parietal lobe to auditory spatial working memory J Cogn Neurosci 2008 20 285 295 10.1162/jocn.2008.20014 18275335

[B14] Lee JH Van der De Groot V Beckerman H Wagenaar RC Lankhorst GJ Bouter LM The intra- and interrater reliability of the action research arm test: a practical test of upper extremity function in patients with stroke Arch Phys Med Rehabil 2001 82 14 19 10.1053/apmr.2001.18668 11239280

[B15] Platz T Pinkowski C van Wijck F Kim IH di Bella P Johnson G Reliability and validity of arm function assessment with standardized guidelines for the Fugl-Meyer Test, Action Research Arm Test and Box and Block Test: a multicentre study Clin Rehabil 2005 19 404 411 10.1191/0269215505cr832oa 15929509

[B16] Wade DT Collin C The Barthel ADL Index: a standard measure of physical disability? Int Disabil Stud 1988 10 64 67 3042746 10.3109/09638288809164105

[B17] Goodglass H Kaplan E Boston diagnostic aphasia examination 1983 Philidelphia: Williams & Wilkins

[B18] Gauthier L Dehaut F Joanett J The Bell Test: A quantitative and qualitative test for visual neglect International Journal of Clinical Neuropsychology 1989 11 49 54

[B19] Group I A S I 3D audio rendering and evaluation guidelines 1998 Los Angeles CA: MIDI Manufacturers Association Incorporated

[B20] Cirstea MC Levin MF Compensatory strategies for reaching in stroke Brain 2000 123 Pt 5 940 953 10.1093/brain/123.5.940 10775539

[B21] Roby-Brami A Fuchs S Mokhtari M Bussel B Reaching and grasping strategies in hemiparetic patients Motor Control 1997 1 72 91

[B22] Boulinguez P Nougier V Velay JL Manual asymmetries in reaching movement control. I: Study of right-handers Cortex 2001 37 101 122 10.1016/S0010-9452(08)70561-6 11292156

[B23] Hodges NJ Lyons J Cockell D Reed A Elliott D Hand, space and attentional asymmetries in goal-directed manual aiming Cortex 1997 33 251 269 10.1016/S0010-9452(08)70003-0 9220257

[B24] Desmurget M Jordan M Prablanc C Jeannerod M Constrained and unconstrained movements involve different control strategies J Neurophysiol 1997 77 1644 1650 9084629 10.1152/jn.1997.77.3.1644

[B25] Boulinguez P Velay JL Nougier V Manual asymmetries in reaching movement control. II: Study of left-handers Cortex 2001 37 123 138 10.1016/S0010-9452(08)70562-8 11292158

[B26] Winstein CJ Pohl PS Effects of unilateral brain damage on the control of goal-directed hand movements Exp Brain Res 1995 105 163 174 10.1007/BF00242191 7589312

[B27] Bagesteiro LB Sainburg RL Nondominant arm advantages in load compensation during rapid elbow joint movements J Neurophysiol 2003 90 1503 1513 10.1152/jn.00189.2003 12736237 PMC10704424

[B28] Haaland KY Schaefer SY Knight RT Adair J Magalhaes A Sadek J Sainburg RL Ipsilesional trajectory control is related to contralesional arm paralysis after left hemisphere damage Exp Brain Res 2009 196 2 195 204 10.1007/s00221-009-1836-z 19479246 PMC2693774

[B29] Bagesteiro LB Sainburg RL Handedness: dominant arm advantages in control of limb dynamics J Neurophysiol 2002 88 2408 2421 10.1152/jn.00901.2001 12424282 PMC10709816

[B30] Haaland KY Prestopnik JL Knight RT Lee RR Hemispheric asymmetries for kinematic and positional aspects of reaching Brain 2004 127 1145 1158 10.1093/brain/awh133 15033898

[B31] Haaland KY Harrington DL Knight RT Spatial deficits in ideomotor limb apraxia. A kinematic analysis of aiming movements Brain 1999 122 Pt 6 1169 1182 10.1093/brain/122.6.1169 10356068

[B32] Hermsdorfer J Blankenfeld H Goldenberg G The dependence of ipsilesional aiming deficits on task demands, lesioned hemisphere, and apraxia Neuropsychologia 2003 41 1628 1643 10.1016/S0028-3932(03)00097-6 12887988

[B33] Saygin AP Dick F Wilson SM Dronkers NF Bates E Neural resources for processing language and environmental sounds: evidence from aphasia Brain 2003 126 928 945 10.1093/brain/awg082 12615649

[B34] Ducommun CY Murray MM Thut G Bellmann A Viaud-Delmon I Clarke S Michel CM Segregated processing of auditory motion and auditory location: an ERP mapping study Neuroimage 2002 16 76 88 10.1006/nimg.2002.1062 11969319

[B35] Adriani M Maeder P Meuli R Thiran AB Frischknecht R Villemure JG Mayer J Annoni JM Bogousslavsky J Fornari E Thiran JP Clarke S Sound recognition and localization in man: specialized cortical networks and effects of acute circumscribed lesions Exp Brain Res 2003 153 591 604 10.1007/s00221-003-1616-0 14504861

[B36] Hyndman D Pickering RM Ashburn A The influence of attention deficits on functional recovery post stroke during the first 12 months after discharge from hospital J Neurol Neurosurg Psychiatry 2008 79 656 663 10.1136/jnnp.2007.125609 17872979

[B37] Keulen RF Adam JJ Fischer MH Kuipers H Jolles J Distractor interference in selective reaching: effects of hemispace, movement direction, and type of movement Cortex 2007 43 531 541 10.1016/S0010-9452(08)70247-8 17623999

[B38] Hoellinger T Auvray M Roby-Brami A Hanneton S Localisation tasks with a three-dimensional audio-motor coupling based on an electromagnetic motion capture device International Multisensory Research Forum: July 16-19. Hamburg, Germany 2008

[B39] Pisella L Grea H Tilikete C Vighetto A Desmurget M Rode G Boisson D Rossetti Y An 'automatic pilot' for the hand in human posterior parietal cortex: toward reinterpreting optic ataxia Nat Neurosci 2000 3 729 736 10.1038/76694 10862707

[B40] Dozza M Horak FB Chiari L Auditory biofeedback substitutes for loss of sensory information in maintaining stance Exp Brain Res 2007 178 37 48 10.1007/s00221-006-0709-y 17021893

[B41] Wolpert DM Ghahramani Z Jordan MI Are arm trajectories planned in kinematic or dynamic coordinates? An adaptation study Exp Brain Res 1995 103 460 470 10.1007/BF00241505 7789452

